# Impact of Dietary Carbohydrate Levels on Growth Performance, Feed Efficiency, and Immune Response in *Litopenaeus vannamei* Cultured in Biofloc Systems

**DOI:** 10.3390/metabo15060409

**Published:** 2025-06-17

**Authors:** Yulong Sun, Shuailiang Zhang, Wenping Feng, Yunqi Zhang, Tao Han, Jiteng Wang

**Affiliations:** Department of Aquaculture, Zhejiang Ocean University, Zhoushan 316022, China; 2022214@zjou.edu.cn (Y.S.); fengwp@zjou.edu.cn (W.F.);

**Keywords:** biofloc technology, *Litopenaeus vannamei*, carbohydrate levels, oxidative stress, immune response

## Abstract

**Background/Objective:** Over an eight-week period, this study assessed the influence of dietary carbohydrate levels on growth, metabolism, and immunity in Pacific white shrimp (*Litopenaeus vannamei*) raised within a biofloc technology (BFT) system. **Methods:** Five isonitrogenous and isolipidic diets, spanning carbohydrate levels from 11% to 47%, were evaluated. **Results:** The results showed that dietary carbohydrate significantly impacted both growth performance and feed utilization. The diet containing 38% carbohydrate yielded the best outcomes, resulting in the highest weight gain, specific growth rate, and an optimal feed conversion ratio in the shrimp. Hepatopancreatic metabolic analysis revealed that the shrimp adapted to diets high in carbohydrates through the upregulation of glycolytic enzymes (PK, PFK) and downregulation of gluconeogenic enzymes (PEPCK, G6Pase). By optimizing the water quality and supplementing microbial nutrition, *L. vannamei* in the BFT system exhibited enhanced dietary carbohydrate utilization and strengthened innate immunity. Specifically, SOD and CAT activities remained largely unaffected by varying carbohydrate levels. However, excessive carbohydrate intake still induced oxidative stress. The high-sugar group (47%) exhibited a significant increase in hemolymph MDA content (*p* < 0.05), with corresponding metabolic alterations observed in glucose, triglyceride, and total protein levels. On the basis of the results of this study, the BFT system may mitigate the adverse effects of a high-carbohydrate diet by enhancing lysosomal enzyme activity (e.g., ACP) and increasing total protein levels. **Conclusions:** These findings suggest that the BFT system enhances shrimp immunity and mitigates the potential adverse effects of imbalanced dietary components. Piecewise regression analysis determined the optimal dietary carbohydrate level for shrimp within the BFT system to be 31.44–31.77%.

## 1. Introduction

The Pacific white shrimp (*Litopenaeus vannamei*), native to Central and South America, is one of the primary aquaculture species in China, highly valued for its rapid growth and exceptional adaptability. In recent years, particularly by 2024, its production has accounted for over 80% of China’s total shrimp production, establishing its dominance in the industry (China Fishery Statistical Yearbook, 2024). However, with the advancement of large-scale and high-density shrimp aquaculture, water pollution and disease risks have become increasingly prominent, posing severe challenges to the development of the entire industry [1]. Excessive ammonia and nitrite—primarily derived from uneaten feed and shrimp waste—pose a significant threat to shrimp health due to the toxicity of ammonia nitrogen after prolonged accumulation [2]. Traditional water quality management methods often rely on frequent water exchanges and chemical treatments, which not only lead to increased costs but also easily trigger environmental pollution problems, contradicting the concept of sustainable and green aquaculture. Therefore, these contradictions exposed in intensive aquaculture systems urgently need to be addressed, necessitating the exploration of novel, environmentally friendly, and efficient aquaculture models [3].

Biofloc technology (BFT) offers an ecologically responsible aquaculture approach centered on microbial management, thereby presenting substantial potential for improving aquaculture practices [4]. At its core, BFT involves strategically adjusting the water’s carbon-to-nitrogen (C/N) ratio to promote synergistic interactions between heterotrophic microorganisms and nitrifying bacteria, which effectively convert residual feed and metabolic byproducts into protein-rich bioflocs [5], providing essential compounds such as lipids and amino acids to shrimp [6]. This conversion process enables a zero-water exchange system while maintaining optimal water quality [7]. Furthermore, BFT not only supplies supplemental nutrition—providing proteins, lipids, minerals, and vitamins [6,8,9,10,11]—but also enhances digestive and antioxidant enzyme activities in cultured organisms, thereby supporting their overall growth and health [12,13].

Proteins, lipids, and carbohydrates represent the primary energy sources in aquaculture feeds. Notably, carbohydrates are generally considered to be more readily available and cost-effective than proteins and lipids. Their inclusion in feed formulations not only helps stabilize pellet structure but also provides an alternative energy source, thereby reducing reliance on lipids or proteins and potentially lessening feed oxidation as well as decreasing ammonia emissions. Moreover, although shrimp feeds typically incorporate carbohydrates at 20–30%, the optimal inclusion level is affected by multiple factors, such as the carbohydrate source [14], shrimp size, feeding strategies [15], and the overall culture environment [16]. For example, Cardona et al. [17] reported that natural food accounted for 37–40% of the total intake in juvenile blue shrimp (*Litopenaeus stylirostris*) reared in a BFT system with formulated feed, and similarly, Zhu et al. [18] found that molasses supplementation in a giant freshwater prawn (*Macrobrachium rosenbergii*) pond culture did not compromise growth, even when feed protein levels were reduced.

While carbohydrate requirements in *L. vannamei* feeds have been investigated under traditional mariculture conditions, the applicability of these findings to BFT systems remains unclear. For example, Guo et al. [19] suggested an optimal corn starch level of 10–20% for *L. vannamei* (0.96 g) at a 38% protein level, while Hu et al. [20] proposed a minimum carbohydrate inclusion level of 19.41% to spare protein. Given the documented differences in nutritional requirements between BFT and traditional aquaculture and the absence of data on dietary carbohydrate needs for *L. vannamei* within BFT systems, this study aims to investigate the effects of varying dietary carbohydrate levels on growth performance, feed utilization, carbohydrate metabolism enzyme activity, and immunity of *L. vannamei* under BFT conditions. The findings will provide theoretical guidance for developing optimized BFT feeds and offer insights into enhancing shrimp feed formulations, improving aquaculture efficiency, and formulating low-nitrogen, environmentally responsible feeds.

## 2. Materials and Methods

### 2.1. Experimental Diets

In this study, corn starch was used as the carbohydrate source. With reference to previous research [1,2], five isonitrogenous and isolipidic diets with varying carbohydrate levels (11%, 20%, 29%, 38%, and 47%) were prepared (Table 1). The diet preparation process was as follows. First, all dry ingredients were sieved and finely ground using a grinder and then mixed at medium speed for approximately 15 min using a mixer to ensure homogeneity. Subsequently, the calculated amounts of lipids and an appropriate amount of distilled water were added sequentially according to the predetermined ratios until the mixture reached approximately 30% wet weight. The mixture was then thoroughly stirred until a homogenous dough formed, followed by a 15 min resting period to allow for even moisture distribution. The dough was then fed into a twin-screw extruder with a 1.0 mm die (model G-250, South China University of Technology, Guangzhou, China). The equipment was operated under controlled temperature and a stable speed to ensure uniform feed pellet formation. The formed feed was initially cooled at room temperature and then dried in a temperature-controlled oven at 45 °C for 24 h to effectively remove moisture, minimize thermal damage to nutrients, prevent oxidation, and ensure stability. After drying, all diets were quickly sealed and packaged and stored at −20 °C to prevent microbial growth and nutrient degradation, ensuring that the feed quality remained stable throughout the experiment.

### 2.2. Culture Experiment

Pacific white shrimp juveniles (*L. vannamei*) were obtained from Zhejiang Yize Aquaculture Co., Ltd. (Taizhou, China) and transported to 1-ton tanks at Zhejiang Ocean University in 100 L of source pond water. Upon arrival, the shrimp were acclimated for 14 days in filtered seawater (salinity: 29 PSU) under biofloc technology conditions. Throughout the acclimation period, the shrimp received feed three times daily (08:30, 16:30, and 23:00), with sucrose supplementation to sustain a carbon-to-nitrogen (C: N) ratio of 20:1 [21]. A total of 300 Pacific white shrimp juveniles (0.79 ± 0.01 g), acclimated for 14 days, were randomly distributed across 15 high-density polyethylene tanks (120 L, 20 shrimp/tank) to establish five experimental groups (n = 3 replicates per group). A biofloc technology (BFT) system with zero water exchange was employed; water lost to evaporation was replaced weekly with aerated tap water. Initial conditions included 8 mL/L biofloc in 30 L of water to encourage robust biofloc formation. Seawater was then added in 10 L increments each week until a total volume of 100 L was achieved, a process designed to balance rapid biofloc propagation with optimal volume and stocking density. Biofloc levels were maintained below 15 mL/L by removing excess. Over the eight-week (54-day) BFT culture, the thermal growth coefficient (TGC) was used to predict weight gain and guide feed input adjustments for stable nitrogen levels. Feeding occurred thrice daily (08:30, 16:30, 23:00), with feeding rates adjusted weekly based on shrimp weight.

### 2.3. Water Quality Parameter Measurement

Water quality parameters were systematically monitored throughout the eight-week experiment using multiple methods to ensure data comprehensiveness and accuracy. First, total ammonia nitrogen (TAN) in water samples was determined using the salicylate method [22] in conjunction with a multi-parameter portable water quality analyzer (LH-M900) to assess whether ammonia nitrogen concentrations were maintained within an appropriate range. Second, nitrite and nitrate concentrations were determined using ion chromatography [23], following the methods of Zhang et al. (2025) [24], to accurately reflect nitrogen transformation in the water. Furthermore, biofloc floc volume (FV) was measured weekly using an Imhoff cone, and the solids ratio was calculated after filtering and drying 1 L water samples to a constant weight, further quantifying the total suspended solids (TSS) [24] to ensure that the experimental data adequately reflected the actual conditions of the aquaculture water.

### 2.4. Sample Collection

After an eight-week (54-day) biofloc system culture period, the shrimp were subjected to a 24 h fasting period prior to weighing. From each tank, eight shrimp were randomly selected and anesthetized on ice, and their hemolymph was carefully extracted using sterile 1.0 mL syringes and then transferred into 1.5 mL anticoagulant tubes (containing EDTA-K2 as the anticoagulant; Labshark, Hunan BKMAM Holding Co., Ltd., Changde, China) under aseptic conditions. The collected hemolymph was subsequently centrifuged at 5000× *g* for 10 min at 4 °C, after which, the supernatant was stored at −80 °C for subsequent hematological analyses. For assessment of shrimp composition, four individuals per replicate tank were randomly selected and stored at −20 °C. Hepatopancreas samples were dissected from the remaining shrimp, immediately frozen in liquid nitrogen, and stored at −80 °C, pending enzyme activity determination. Biofloc samples were obtained from the culture water by filtration (48 μm sieve) and stored at −20 °C until analyzed for proximate constituents.

### 2.5. Proximate Composition Analysis

Following established methods [25], feed, whole shrimp, and biofloc samples were analyzed for crude protein, crude lipid, moisture, and ash. Moisture content was determined gravimetrically by measuring the weight loss of the samples after freeze-drying (LL1500, Thermo, Waltham, Massachusetts, USA) to a constant mass. Crude protein was quantified via Dumas combustion using a Dumatec 8000 (FOSS, Hillerød, Denmark) analyzer, with the results calibrated against EDTA and a nitrogen-to-protein conversion factor of 6.25. Crude lipid was extracted from dried samples using organic solvents in a Soxhlet apparatus (E816), with the lipid content determined from the mass of the residue remaining after solvent evaporation. Gross energy was measured by complete combustion of dried samples in a calorimeter (ZDHW-9000DH, Hebi, China ), with the heat released calculated from temperature changes. Ash content was determined by combusting samples in a muffle furnace at 550 °C, and the ash content was calculated from the mass of the residual ash. All analyses were performed with repeated measurements to ensure data reliability

### 2.6. Hepatopancreas and Hemolymph Parameter Measurement

Hepatopancreas samples (0.1 g per sample) were homogenized in 900 µL of 0.9% NaCl solution to yield a 10% (*w*/*v*) homogenate. The homogenate was then centrifuged at 3000 rpm for 10 min at 4 °C, and the resulting supernatant was collected for enzyme activity assays. Enzyme activities and antioxidant parameters in hemolymph and hepatopancreas samples were determined following the protocols of Zhang et al. (2025) [24]. In brief, commercially available assay kits were employed, with all procedures strictly conducted according to the kit instructions. Hemolymph was assayed for glucose (GLU), triglycerides (TG), total cholesterol (T-CHO), total protein (TP), acid phosphatase (ACP), and alkaline phosphatase (ALP). The activities of carbohydrate metabolic enzymes—amylase (AMS), pyruvate kinase (PK), phosphofructokinase (PFK), phosphoenolpyruvate carboxykinase (PEPCK), and glucose-6-phosphatase (G6Pase)—along with the antioxidant markers catalase (CAT), superoxide dismutase (SOD), malondialdehyde (MDA), and total antioxidant capacity (T-AOC) were quantified in hepatopancreas tissue.

### 2.7. Statistical Analysis

To determine statistical significance, the data were initially screened for normality (Kolmogorov–Smirnov test) and homogeneity of variance (Levene’s test). If the data met the assumptions of normality and homogeneity, one-way ANOVA was used. Otherwise, the Kruskal–Wallis test was applied. Duncan’s multiple range test was used for post hoc comparisons, and significance was assessed at *p* < 0.05. SPSS version 20.0 was used for all analyses.

## 3. Results

### 3.1. Water Quality

**Figure 1:**
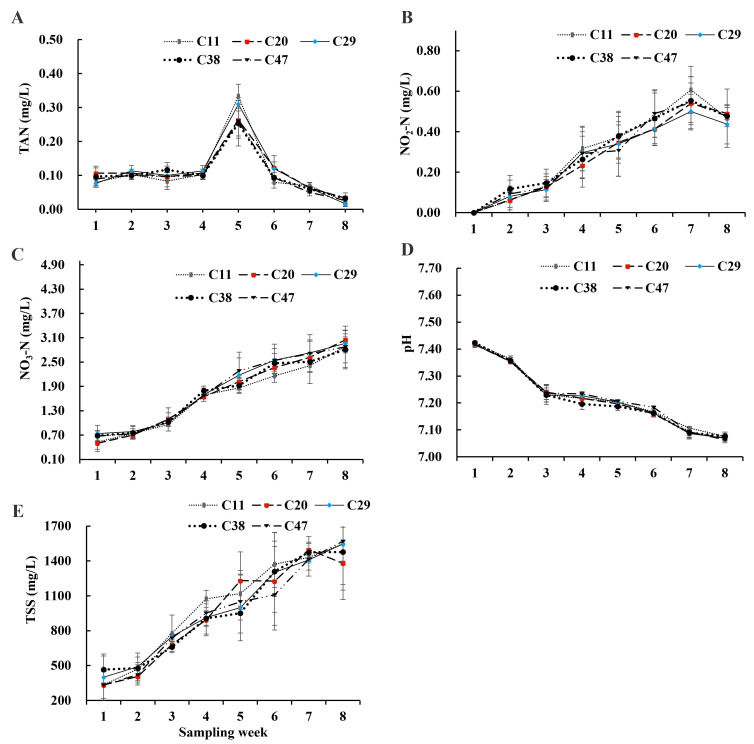
Water quality in the BFT system under varying dietary carbohydrate levels: (**A**) Total ammonia nitrogen (TAN); (**B**) Nitrite (NO_2_^−^); (**C**) Nitrate (NO_3_^−^); (**D**) pH; (**E**) Reduction in total suspended solids (TSS). Data are presented as means ± SD (n = 3).

TAN peaked in week 5 and subsequently declined, remaining below 0.5 mg/L. Nitrite initially increased and then decreased, while nitrate increased over time. TAN, nitrite, and nitrate concentrations were within the suitable range for shrimp culture. pH gradually decreased, while TSS continuously increased (Figure 1).

### 3.2. Growth Performance, Survival, and Feed Utilization

**Figure 2:**
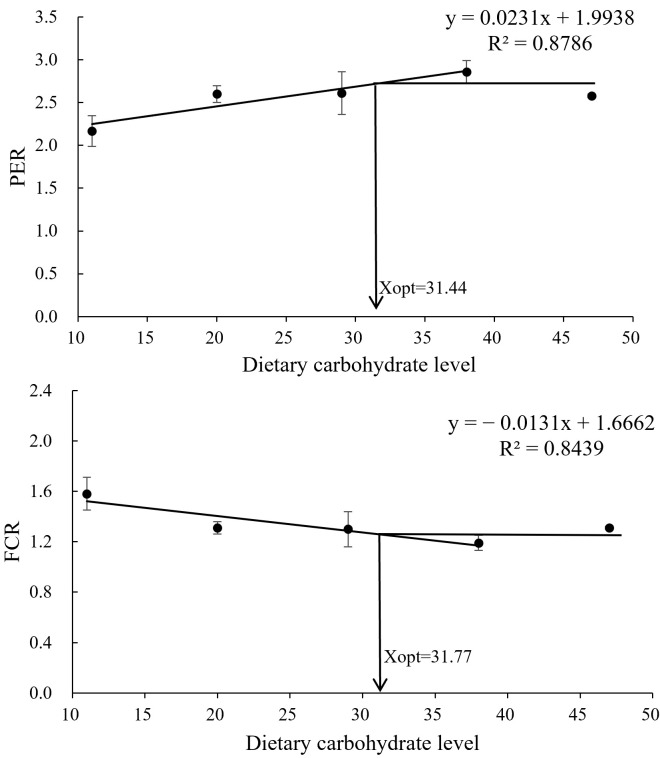
Protein efficiency ratio (PER) and feed conversion ratio (FCR) as functions of dietary carbohydrate levels. “Xopt” indicates the optimal carbohydrate level maximizing PER and FCR in *L. vannamei*. Data are presented as means ± SD (n = 3).

Growth and feed utilization in *L. vannamei* were demonstrably influenced by dietary carbohydrate levels (Table 2). Increasing carbohydrate levels from 11% to 38% significantly enhanced final body weight (FBW), weight gain (WG), and specific growth rate (SGR) (*p* < 0.05). Compared to the other groups, the 11% carbohydrate diet resulted in a significantly elevated feed conversion ratio (FCR) and a reduced protein efficiency ratio (PER). Broken-line regression analysis suggested an optimal dietary carbohydrate range of 31.44–31.77% for *L. vannamei* in BFT systems (Figure 2). Dietary carbohydrate levels did not significantly affect nitrogen utilization (Table 3). Nitrogen retention (NR) increased and then decreased with increasing carbohydrate levels, with the 38% carbohydrate treatment (C38) showing the highest NR and the 11% carbohydrate treatment (C11) showing the lowest. Lipid retention (LR) followed a similar trend, with C38 showing the highest LR, despite the 11% carbohydrate treatment (C11) having significantly higher daily lipid intake (DLI).

### 3.3. Proximate Composition of Shrimp and Biofloc

The influence of dietary carbohydrate levels on both *L. vannamei* body composition and biofloc composition was evaluated. Dietary carbohydrate had no significant effect on shrimp body composition, apart from ash content (*p* > 0.05). Body fat tended to increase with rising carbohydrate concentrations in the diet, although this trend was not statistically significant. Biofloc composition also remained unaffected by the dietary treatments (Table 4).

### 3.4. Hemolymph Biochemical Parameters

**Figure 3:**
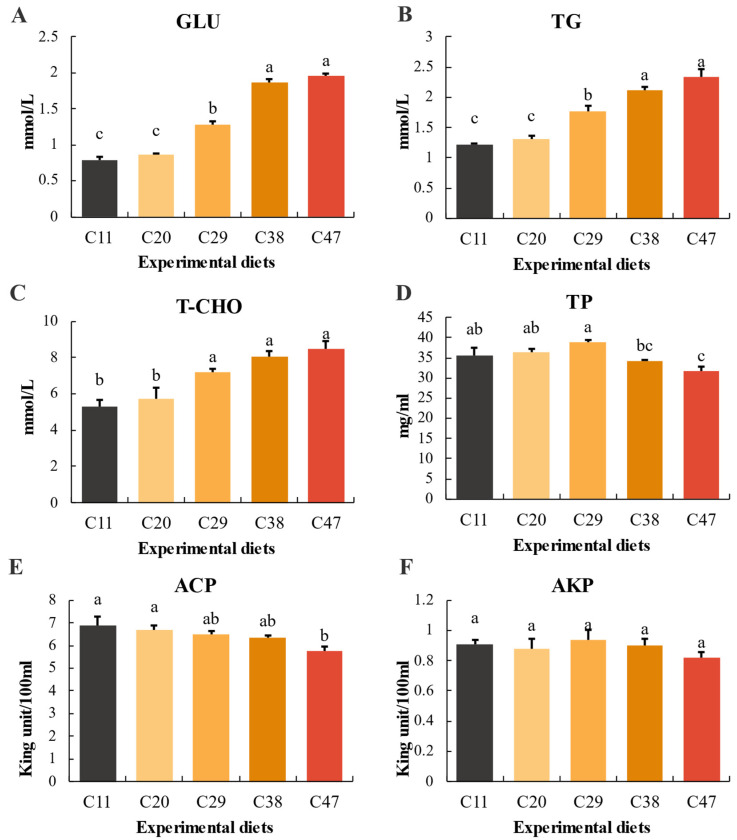
Hematological parameters in *L. vannamei* within the BFT system, as influenced by dietary carbohydrate levels: (**A**) Glucose (GLU); (**B**) Triglycerides (TG); (**C**) Total cholesterol (T-CHO); (**D**) Total protein (TP); (**E**) Acid phosphatase (ACP); (**F**) Alkaline phosphatase (ALP). Data are presented as means ± SD (n = 3), with significant differences among treatments indicated by different letters (*p* < 0.05).

Dietary carbohydrate levels significantly affected *L. vannamei* hemolymph biochemical parameters, except for AKP (*p* < 0.05) (Figure 3). Hemolymph GLU and TG increased with carbohydrate levels, with the C38 and C47 groups significantly higher than the others. T-CHO significantly increased with carbohydrate levels. TP and ACP significantly decreased as carbohydrate levels increased from 11% to 47%. Though not statistically significant, AKP was lowest in the C47 group.

### 3.5. Amylase and Carbohydrate Metabolism Enzyme Activity

**Figure 4:**
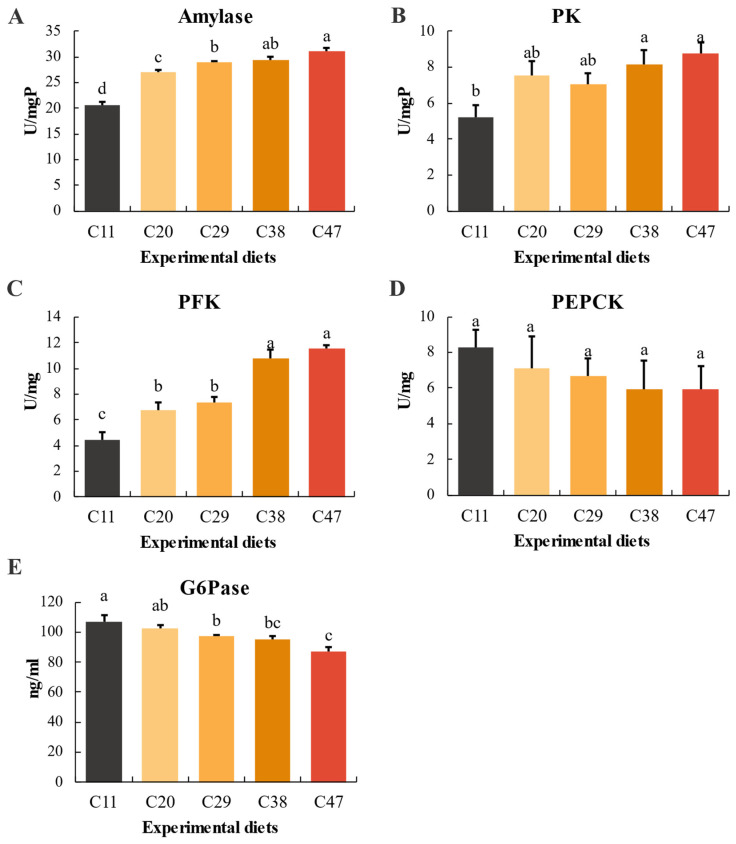
Amylase and carbohydrate metabolic enzyme activities in *L. vannamei* within the BFT system, as influenced by dietary carbohydrate levels: (**A**) Amylase; (**B**) Pyruvate kinase (PK); (**C**) Phosphofructokinase (PFK); (**D**) Phosphoenolpyruvate carboxykinase (PEPCK); (**E**) Glucose-6-phosphatase (G6Pase). Data are presented as means ± SD (n = 3), with significant differences among treatments indicated by different letters (*p* < 0.05).

Figure 4 illustrates the significant impact of dietary carbohydrate levels on enzyme activities in *L. vannamei* (*p* < 0.05). A positive correlation was observed between dietary carbohydrate and amylase activity, with amylase levels increasing significantly with higher carbohydrate content. Conversely, PEPCK and G6Pase activities displayed a negative correlation, decreasing as carbohydrate levels increased. PK and PFK activities also increased significantly, showing a positive trend with carbohydrate levels ranging from 11% to 47% (*p* < 0.05) (Figure 4).

### 3.6. Antioxidant Enzymes

**Figure 5:**
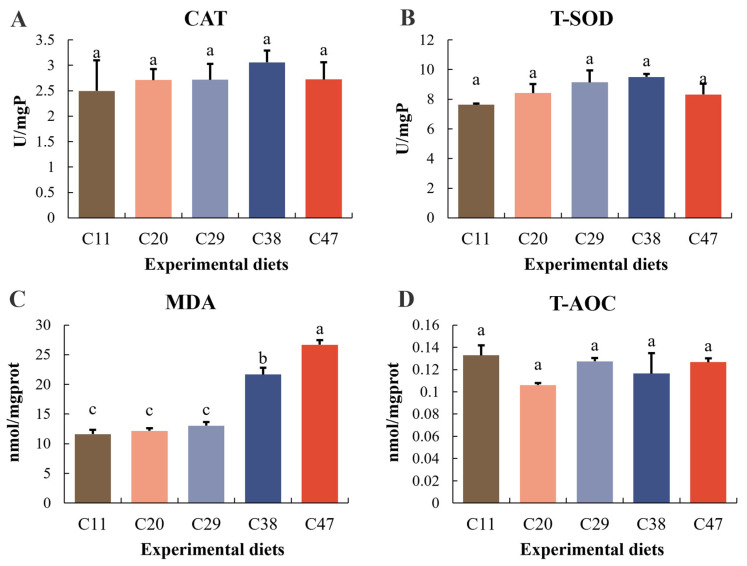
Antioxidant enzyme activity in *L. vannamei* within the BFT system as influenced by dietary carbohydrate levels: (**A**) Catalase (CAT); (**B**) Total superoxide dismutase (T-SOD); (**C**) Malondialdehyde (MDA); (**D**) Total antioxidant capacity (T-AOC). Data are presented as means ± SD (n = 3), with significant differences among treatments indicated by different letters (*p* < 0.05).

Dietary carbohydrate levels impacted specific antioxidant markers in *L. vannamei*. CAT and T-SOD activities initially rose and then fell as carbohydrate levels increased, with peak values observed in the C38 group. MDA levels were also significantly affected by dietary carbohydrate (*p* < 0.05), with the C38 and C47 groups displaying significantly higher MDA. Total antioxidant capacity (T-AOC), however, showed no significant response to varying carbohydrate levels (Figure 5).

## 4. Discussion

Traditional aquaculture relies heavily on water exchange to manage ammonia and nitrite toxicity, maintaining acceptable water quality [26]. BFT systems, conversely, operate by adding a carbon source, enabling heterotrophic bacteria to convert nitrogenous compounds into microbial biomass [27], sustaining suitable water quality with zero water exchange [3,27,28]. Generally, BFT systems for *L. vannamei* should maintain ammonia and nitrite concentrations below 1 mg/L and nitrate between 0.5 and 20 mg/L [29]. In this study, TAN, nitrite, and nitrate were within these ranges. Total suspended solids, a key indicator of BFT development [27,30], increased during the experiment, indicating effective BFT development following carbon addition. The pH gradually decreased, potentially from bacterial activity [31], as increased heterotrophic respiration elevates water CO_2_, lowering pH [32,33,34]. However, pH remained suitable (6.8–8.0), supporting shrimp growth.

Beyond water quality, BFT systems provide supplemental nutrition. Marine and freshwater shrimp readily consume BFT as a natural food source [35]. Cardona et al. [17] reported that natural food accounted for 37–40% of intake in juvenile blue shrimp (*L. stylirostris*) in BFT systems. Although carbohydrate levels did not significantly alter BFT composition, BFT contained substantial protein (26.97–33.40%) and lipids (1.21–1.44%), providing valuable nutrition for *L. vannamei*. In the BFT system, *L. vannamei* displayed optimal growth and feed utilization at 38% dietary carbohydrate (C38). WG and SGR were significantly greater in C38 than in C11 and C20 but did not differ from C29 and C47. Broken-line regression estimated an optimal dietary carbohydrate level of 31.44–31.77% in the BFT system.

No studies have directly examined carbohydrate requirements of *L. vannamei* feed in BFT systems. In traditional mariculture, Guo et al. [19] found 10–20% corn starch optimal for juvenile *L. vannamei* with 38% dietary protein, based on weight gain, survival, FCR, and digestibility. This is lower than our results, suggesting enhanced carbohydrate utilization in BFT systems. A previous study in our lab also indicated a higher suitable carbohydrate-to-lipid ratio in BFT systems [24]. However, 47% carbohydrate reduced growth and feed utilization, similar to observations in traditional systems where growth declines and FCR increases above 25% dietary starch [19]. Excessive carbohydrate intake has been shown to negatively affect growth and feed utilization in various aquatic species, including red-spotted grouper (*Epinephelus akaara*) [36], golden pompano (*Trachinotus ovatus*) [37], blackmouth croaker (*Nibea japonica*) [38], and sea cucumber (*Apostichopus japonicus*) [39]. In our study, the lower WG, SGR, PER, and NR values along with the higher FCR observed in the C11 group suggest that insufficient dietary carbohydrate may hinder growth performance and feed efficiency. Consistently, Hu et al. [20] reported that a dietary carbohydrate level of 13.82% was inadequate for optimal growth in *L. vannamei*. Moreover, low carbohydrate intake and reduced energy availability have also been associated with restricted growth and impaired feed utilization in mud crab (*Scylla paramamosain*) [40]. Within the carbohydrate range of 11–38%, our findings indicate a positive correlation between dietary carbohydrate levels and *L. vannamei* growth performance, feed utilization efficiency, PER, and NR, highlighting the importance of optimizing carbohydrate intake for sustainable shrimp culture. Appropriate carbohydrate supplementation, serving as a non-protein energy source, not only enhances growth but also helps spare protein for other physiological functions. Similarly, studies on mud crab have demonstrated that dietary carbohydrate levels between 6% and 24% improve both growth and protein utilization efficiency [40]. These findings highlight the importance of balanced carbohydrate inclusion in aquafeeds, enhancing nutrient utilization and reducing protein dependence.

Shrimp amylase effectively digests starch, releasing glucose that is utilized with high efficiency [41,42,43]. Amylase activity is directly correlated with the dietary carbohydrate level, a trend also observed in *L. vannamei* [24], razor clam (*Sinonovacula constricta*) [44], and Caspian roach (*Rutilus frisii*) [45]. In crustaceans, the hepatopancreas is the principal organ responsible for regulating carbohydrate metabolism, employing both glycolysis and gluconeogenesis to maintain hemolymph glucose homeostasis. The key glycolytic enzymes PFK and PK facilitate the breakdown of glucose for energy production [46]. In this study, elevated PK and PFK activities accompanied increased carbohydrate levels, indicating that *L. vannamei* compensates for high-carbohydrate diets by enhancing its glycolytic capacity. Similarly, elevated dietary carbohydrate levels have been shown to boost glycolytic enzyme activities in Japanese freshwater prawn (*Macrobrachium nipponense*) [47] and to increase both PK and PFK activities in grouper [48], consistent with our previous findings on carbohydrate-to-lipid ratios in *L. vannamei* in BFT systems [24]. Conversely, the key gluconeogenic enzymes G6Pase and PEPCK, crucial for stabilizing hemolymph glucose levels [49,50], were suppressed as carbohydrate levels increased. This suppression aligns with observations of dietary carbohydrate-induced inhibition of gluconeogenesis in red swamp crayfish [51] and largemouth bass (*Micropterus salmoides*) [52].

Hemolymph biochemical parameters reflect nutritional metabolism and health in *L. vannamei*. Carbohydrate levels significantly influenced hemolymph GLU, TG, and T-CHO. Hemolymph glucose provides energy, and blood glucose peaks correlate with digestible carbohydrate [53,54]. TG and T-CHO, lipid forms transported in blood, reflect lipid metabolism [55]. In this experiment, *L. vannamei* hemolymph GLU, TG, and T-CHO increased with carbohydrate, with the C38 and C47 groups higher, suggesting more active glucose and lipid transport with higher carbohydrate. This aligns with previous studies in orange-spotted grouper (*Epinephelus coioides*) [55], golden pompano (*Trachinotus ovatus*) [37], and Nile tilapia (*Oreochromis niloticus*) [56]. Excessive carbohydrate intake can lead to fat deposition [57]. While statistically insignificant, *L. vannamei* body fat and LR increased with carbohydrate, as lipid catabolism is often hindered when glucose is abundant, leading to tissue deposition [58,59].

Antioxidant capacity indicates invertebrate health [60]. ROS imbalances can trigger oxidative stress [61]. SOD reduces oxidative damage [62], while CAT further decomposes hydrogen peroxide [63]; they act synergistically. In this study, dietary carbohydrate did not significantly affect SOD and CAT in *L. vannamei*. Hamidoghli et al. showed *L. vannamei* SOD was not significantly affected by lipid from 4.5% to 15%, possibly because BFT systems enhance shrimp innate immunity, increasing their antioxidant capacity and lessening the impact of lipid levels [64]. Our results are similar to previous findings on carbohydrate-to-lipid ratios in Pacific white shrimp within BFT systems [24], indicating that appropriate carbohydrate levels within BFT systems enhance SOD and CAT activities, thereby partially mitigating the adverse effects of starch. Although SOD and CAT activities in the C38 group were numerically higher, the differences were not statistically significant. Furthermore, Wu et al. [65] found that a dietary carbohydrate content of 28.84% resulted in optimal growth performance and CAT and SOD activities in black carp (*Mylopharyngodon piceus*), demonstrating that moderate carbohydrate levels can activate the immune system, with moderate levels of ROS positively regulating immune function [66]. Combined with the results of this study, this suggests that BFT systems can, to some extent, alleviate the negative effects of high carbohydrate levels.

However, long-term, high-level carbohydrate intake can still lead to excessive ROS accumulation in the body, thereby impairing the immune system. MDA is produced during lipid peroxidation and is a common indicator of oxidative damage within the body [67]. Our results indicate that MDA was significantly higher in the C47 group compared to the other groups, further demonstrating that long-term, high-carbohydrate intake readily induces oxidative stress [68], a phenomenon also reported in largemouth bass (*Micropterus salmoides*) [52,69], Japanese freshwater prawn (*Macrobrachium nipponense*) [47], and snakehead (*Channa argus*) [70]. Additionally, our results showed a decrease in WG and SGR and an increase in FCR in the C47 group compared to the C38 group, although not statistically significant. Previous studies have indicated that ingesting inappropriate carbohydrate ratios or prolonged exposure to high-carbohydrate environments exceeding the metabolic threshold can disrupt physiological metabolic balance, adversely affecting growth, health, and protein deposition, ultimately reducing growth performance and feed efficiency [51,54,71,72]. The significantly elevated MDA levels in the C47 group further corroborate that excessive carbohydrate intake exacerbates stress in farmed aquatic animals. Therefore, although BFT systems partially alleviate the adverse effects of high carbohydrate levels by enhancing microbial activity and promoting inorganic nitrogen fixation, these compensatory mechanisms are often insufficient to fully offset the oxidative burden caused by long-term, excessive carbohydrate intake.

ACP and AKP are key components of lysosomal enzymes and play important roles in cellular immunity [73]. These are often measured to assess immunity [74]. In this study, hemolymph ACP was significantly lower in C47, and hemolymph AKP was also lowest, though not significantly, in C47. Zhang et al. [66] found that higher dietary carbohydrate (25–30%) decreased serum AKP in brook trout (*Salvelinus fontinalis*), and AKP also decreased with high-carbohydrate diets in largemouth bass [69]. Wen et al. [75] found lower ACP activity in *Procambarus clarkii* with 25% protein and 39% carbohydrate. Furthermore, C47 exhibited the lowest hemolymph total protein (TP) levels, which are related to immunity [76], indicating that high-carbohydrate diets may compromise the immunocompetence of *L. vannamei*. This finding further reinforces previous inferences that, while BFT systems can partially alleviate the negative effects of high carbohydrate levels, they are often insufficient to fully offset the physiological stress induced by long-term, excessive carbohydrate intake. Therefore, carefully controlling carbohydrate intake levels remains a critical factor in optimizing culture strategies and improving individual health and immunity within BFT aquaculture systems.

While the findings of this study suggest that the BFT system may enhance *L. vannamei*’s ability to utilize dietary carbohydrates and improve innate immunity; however, certain limitations should be acknowledged. The use of three replicates per treatment (n = 3) may have reduced statistical power and increased the risk of false negatives or false positives, particularly for highly variable biochemical parameters. Future studies should incorporate a greater number of biological replicates to improve the validation of the results. Furthermore, biofloc characterization in this study was limited to proximate composition. Advanced techniques, such as metagenomics, could provide a more comprehensive microbial and functional profile, offering deeper insights into its role in nutrient utilization and immune modulation under varying carbohydrate regimes.

Given that feed costs constitute a significant proportion of expenses in intensive shrimp aquaculture [4,77] and that biofloc technology (BFT) can substantially reduce water exchange requirements [3,7], it offers a means to further control operational costs. BFT systems are increasingly adopted in commercial aquaculture due to their advantages, including promoted nutrient cycling [78,79], increased production efficiency, reduced farming costs [80], minimized environmental impact, and decreased disease incidence [4,81,82]. However, challenges remain that necessitate further research, such as optimizing specialized feed formulations, standardizing management protocols, and implementing proper biofloc disposal strategies at the end of the production cycle.

## 5. Conclusions

*L. vannamei* exhibits enhanced carbohydrate utilization in BFT systems compared to traditional aquaculture. It upregulates glycolysis and downregulates gluconeogenesis, and increased dietary carbohydrate within limits enhances growth and spares protein. BFT systems enhance inherent immunity, mitigating some effects of varying carbohydrate; however, prolonged high-carbohydrate diets still cause oxidative stress. Optimal dietary carbohydrate content is 31.44–31.77%.

## Figures and Tables

**Table 1:** Experimental diet composition and proximate composition (% dry matter).

Composition (%)	Dietary Carbohydrate Level				
	11	20	29	38	47
^a^ Fish meal	39.51	39.51	39.51	39.51	39.51
**Corn starch**	11.00	20.00	29.00	38.00	47.00
Fish oil	1.11	1.11	1.11	1.11	1.11
Soybean oil	2.59	2.59	2.59	2.59	2.59
Phospholipid	1.00	1.00	1.00	1.00	1.00
Cholesterol	0.60	0.60	0.60	0.60	0.60
^b^ Vitamin mix	1.80	1.80	1.80	1.80	1.80
Choline chloride	0.30	0.30	0.30	0.30	0.30
^c^ Mineral mix	0.50	0.50	0.50	0.50	0.50
Calcium dihydrogen phosphate	1.50	1.50	1.50	1.50	1.50
Cellulose	37.09	28.09	19.09	10.09	1.09
Taurine	1.00	1.00	1.00	1.00	1.00
Sodium alginate	2.00	2.00	2.00	2.00	2.00
Proximate composition (%)					
Crude protein	29.32	29.42	29.67	29.20	29.44
Crude lipid	8.13	8.11	7.93	8.02	8.29
Moisture	11.73	10.36	11.03	11.20	9.67
Ash	6.81	6.58	6.59	6.34	6.59
^d^ Energy (kJ g^−1^)	12.02	13.59	15.12	16.60	18.33

**^a^**: Purchased from American Seafood Company. **^b^**: Premix composition (g/100 g): Cobalt chloride, 0.004; Copper sulfate pentahydrate, 0.250; Ferrous sulfate, 4.0; Magnesium sulfate heptahydrate, 28.398; Manganese sulfate monohydrate, 0.650; Potassium iodide, 0.067; Sodium selenite, 0.010; Zinc sulfate heptahydrate, 13.193; Cellulose, 53.428. **^c^**: Premix composition (g/kg): Vitamin B1, 0.5; Riboflavin, 3.0; Vitamin B6, 1.0; Calcium pantothenate, 5.0; Niacin, 5.0; Biotin, 0.05; Folic acid, 0.18; Vitamin B12, 0.002; Choline chloride, 100.0; Inositol, 5.0; Menadione, 2.0; Vitamin A (20,000 IU/g), 5.0; Vitamin D3 (400,000 IU/g), 0.002; Vitamin E (250 IU/g), 8.0; Alpha-cellulose, 865.266. **^d^**: The total energy content of the feed was calculated based on protein, lipid, and carbohydrate (starch) at 23.7, 39.5, and 17.2 kJ g⁻^1^, respectively.

**Table 2:** Growth, survival, and feed utilization of *L. vannamei* as influenced by dietary carbohydrate levels in the BFT system.

	Experimental Diet				
	C11	C20	C29	C38	C47
IBW ^1^	0.78 ± 0.01	0.79 ± 0.02	0.79 ± 0.01	0.79 ± 0.01	0.80 ± 0.01
FBW ^2^	5.52 ± 0.27 ^d^	6.88 ± 0.06 ^c^	7.10 ± 0.27 ^bc^	7.78 ± 0.65 ^a^	7.55 ± 0.16 ^ab^
WG ^3^	606.93 ± 35.60 ^c^	773.18 ± 15.66 ^b^	797.60 ± 43.15 ^ab^	887.49 ± 91.13 ^a^	843.67 ± 13.79 ^ab^
SGR ^4^	3.49 ± 0.09 ^c^	3.87 ± 0.03 ^b^	3.92 ± 0.09 ^ab^	4.08 ± 0.16 ^a^	4.01 ± 0.03 ^ab^
FCR ^5^	1.49 ± 0.09 ^a^	1.28 ± 0.06 ^b^	1.28 ± 0.12 ^b^	1.17 ± 0.06 ^b^	1.28 ± 0.02 ^b^
PER ^6^	2.32 ± 0.14 ^a^	2.70 ± 0.14 ^b^	2.72 ± 0.24 ^b^	2.95 ± 0.16 ^b^	2.70 ± 0.05 ^b^
Survival rate (%) ^7^	90.00 ± 5.00	96.67 ± 2.89	93.33 ± 7.64	96.67 ± 5.77	86.67 ± 5.77

Data are presented as mean ± standard deviation (n = 3), with superscript letters indicating significance (a, b, c, d), and significant differences among treatments indicated by differing superscript letters (*p* < 0.05). The absence of superscript letters denotes no significant difference. ^1^ Initial body weight (IBW, g). ^2^ Final body weight (FBW, g). ^3^ Weight gain (WG, %) = 100 × ((FBW − IBW)/IBW. ^4^ Specific growth rate (SGR, %/day) = 100 × (ln (FBW) − ln (IBW))/day. ^5^ Feed conversion ratio (FCR) = Weight gain (g, wet weight)/Feed intake (g, dry weight). ^6^ Protein efficiency ratio (PER) = Wet weight gain/Protein intake. ^7^ Survival rate (%) = 100 × Final shrimp number/Initial shrimp number.

**Table 3:** Nitrogen and lipid utilization in *L. vannamei* in the BFT system as affected by dietary carbohydrate levels.

	Experimental Diet				
	C11	C20	C29	C38	C47
Nitrogen					
DNI ^1^ (g kg ABW^−1^ day^−1^)	1.85 ± 0.11	1.68 ± 0.09	1.69 ± 0.14	1.58 ± 0.06	1.71 ± 0.03
DNG ^2^ (g kg ABW^−1^ day^−1^)	1.07 ± 0.02	1.05 ± 0.04	1.06 ± 0.06	1.04 ± 0.04	1.04 ± 0.02
NR ^3^ (% intake)	57.66 ± 3.80	62.20 ± 3.66	62.81 ± 3.50	65.68 ± 0.12	60.83 ± 1.02
Lipid					
DLI ^4^ (g kg ABW^−1^ day^−1^)	3.25 ± 0.19 ^a^	2.95 ± 0.15 ^bc^	2.89 ± 0.24 ^bc^	2.73 ± 0.10 ^c^	3.06 ± 0.05 ^ab^
DLG ^5^ (g kg ABW^−1^ day^−1^)	0.89 ± 0.01	0.91 ± 0.10	0.89 ± 0.03	0.91 ± 0.15	0.96 ± 0.06
LR ^6^ (% intake)	27.33 ± 1.75	30.90 ± 3.30	31.02 ± 2.07	33.36 ± 4.44	31.25 ± 1.58

Data are presented as mean ± standard deviation (n = 3), with superscript letters indicating significance (a, b, c), and significant differences among treatments indicated by differing superscript letters (*p* < 0.05). The absence of superscript letters denotes no significant difference. Average body weight (ABW, g) = (IBW + FBW)/2. ^1^ Daily nitrogen intake (DNI, g kg⁻^1^ ABW day⁻^1^) = Feed nitrogen intake/ABW × Feeding days. ^2^ Daily nitrogen gain (DNG, g kg⁻^1^ ABW day⁻^1^) = (FBW × Final body nitrogen content − IBW × Initial body nitrogen content)/ABW × Feeding days. ^3^ Nitrogen retention (NR, % intake) = 100 × DNG/DNI. ^4^ Daily lipid intake (DLI, g kg⁻^1^ ABW day⁻^1^) = Feed lipid intake/ABW × Feeding days. ^5^ Daily lipid gain (DLG, g kg⁻^1^ ABW day⁻^1^) = (FBW × Final body lipid content − IBW × Initial body lipid content)/ABW × Feeding days. ^6^ Lipid retention (LR, % intake) = 100 × DLG/DLI.

**Table 4:** Proximate composition (dry matter %) of *L. vannamei* and biofloc from BFT systems as influenced by dietary carbohydrate levels.

	Experimental Diet				
	C11	C20	C29	C38	C47
Whole shrimp					
Moisture (%)	68.24 ± 0.49	68.87 ± 0.91	69.42 ± 0.73	70.58 ± 1.65	69.63 ± 0.21
Crude protein (%)	74.67 ± 0.63	71.76 ± 1.14	73.57 ± 2.77	73.90 ± 0.92	72.33 ± 1.37
Crude lipid (%)	9.72 ± 0.28	9.76 ± 0.83	9.75 ± 0.13	10.17 ± 1.15	10.38 ± 0.59
Ash (%)	13.00 ± 0.43 ^a^	13.03 ± 0.43 ^a^	13.04 ± 0.53 ^a^	12.81 ± 0.65 ^a^	11.81 ± 0.52 ^b^
Biofloc					
Moisture (%)	87.40 ± 0.98	86.15 ± 1.49	86.63 ± 0.94	85.63 ± 0.71	87.38 ± 0.52
Crude protein (%)	30.24 ± 2.68	29.78 ± 1.85	29.45 ± 2.71	28.71 ± 1.15	29.53 ± 3.35
Crude lipid (%)	9.63 ± 0.43 ^ab^	9.11 ± 0.65 ^a^	10.68 ± 0.13 ^b^	10.03 ± 0.87 ^ab^	9.86 ± 0.31 ^ab^
Ash (%)	25.53 ± 0.87	24.95 ± 2.43	25.03 ± 1.60	23.68 ± 2.18	25.74 ± 1.11

Data are presented as mean ± standard deviation (n = 3), with superscript letters indicating significance (a, b, c), and significant differences among treatments indicated by differing superscript letters (*p* < 0.05). The absence of superscript letters denotes no significant difference.

## Data Availability

The data presented in this study are available on request from the corresponding author.
